# Outcome of repaired unstable meniscal tears in children and adolescents

**DOI:** 10.3109/17453674.2012.693017

**Published:** 2012-06-04

**Authors:** Tanja Kraus, Nima Heidari, Martin Švehlík, Frank Schneider, Matthias Sperl, Wolfgang Linhart

**Affiliations:** ^1^Department of Pediatric Orthopaedics, Medical University of Graz, Graz, Austria; ^2^Department of Orthopaedics and Traumatology for Children and Adults, Second Faculty of Medicine, Charles University, Prague, Czech Republic; ^3^Department of Limb Reconstruction, Bristol Royal Infirmary, Bristol, UK; Correspondence: tanja.kraus@medunigraz.at

## Abstract

**Background:**

Unstable meniscal tears are rare injuries in skeletally immature patients. Loss of a meniscus increases the risk of subsequent development of degenerative changes in the knee. This study deals with the outcome of intraarticular meniscal repair and factors that affect healing. Parameters of interest were type and location of the tear and also the influence of simultaneous reconstruction of a ruptured ACL.

**Methods:**

We investigated the outcome of 25 patients (29 menisci) aged 15 (4–17) years who underwent surgery for full thickness meniscal tears, either as isolated lesions or in combination with ACL ruptures. Intraoperative documentation followed the IKDC 2000 standard. Outcome measurements were the Tegner score (pre- and postoperatively) and the Lysholm score (postoperatively) after an average follow-up period of 2.3 years, with postoperative arthroscopy and MRT in some cases.

**Results:**

24 of the 29 meniscal lesions healed (defined as giving an asymptomatic patient) regardless of location or type. 4 patients re-ruptured their menisci (all in the pars intermedia) at an average of 15 months after surgery following a new injury. Mean Lysholm score at follow-up was 95, the Tegner score deteriorated, mean preoperative score: 7.8 (4–10); mean postoperative score: 7.2 (4–10). Patients with simultaneous ACL reconstruction had a better outcome.

**Interpretation:**

All meniscal tears in the skeletally immature patient are amenable to repair. All recurrent meniscal tears in our patients were located in the pars intermedia; the poorer blood supply in this region may give a higher risk of re-rupture. Simultaneous ACL reconstruction appears to benefit the results of meniscal repair.

Isolated meniscal tears in the skeletally immature patient are rare but well-recognized injuries (Hede et al. 1990). Meniscal tears are frequently seen in association with a ruptured ACL (Bellisari et al. 2011, Samora et al. 2011). The menisci reduce contact stress and buffer axial, rotational, and shearing forces, thereby protecting the articular cartilage (Kurosawa et al. 1980). In the classic paper by Fairbank (1948), early osteoarthritis was clearly shown to be the inevitable outcome of total medial menisectomy. This led to the development of various techniques of meniscal repair in order to improve long-term outcome. This is particularly desirable in the immature patient, as early degenerative changes in this population have profound consequences in the long term. There are a variety of all-inside arthroscopic techniques that are relatively easy to master and that can be quick to perform as compared to the more technically demanding inside-out and outside-in methods (Haas et al. 2005, Kotsovolos et al. 2006).

The results of meniscal repair in adults have been reported to be fair to good (Barber 1987, Eggli et al. 1995), while there have been few published data for children and adolescents (Krych et al. 2008). In this retrospective study, we assessed the outcome in a cohort of children and adolescents undergoing meniscal repair and determined whether there was any relationship between the type of meniscal injury and the outcome.

## Material and methods

We conducted a retrospective review of all children and adolescents who had undergone arthroscopic knee surgery over a 2-year period from June 2003 to October 2005, for meniscal lesions at our institution. Patients were identified through the hospital database. Inclusion criteria were: open physes; MRI verification of full-thickness meniscal tear (distorted or not) with or without a concomitant anterior cruciate ligament (ACL) rupture; availability of pre- and postoperative Tegner score (Tegner and Lysholm 1985) and postoperative Lysholm score (Lysholm and Gillquist 1982); and complete International Knee Documentation Committee intraoperative documentation (IKDC 2000) (Hefti et al. 1993, Schmitt et al. 2010). A meniscal tear was diagnosed in the presence of meniscal distortion or if the intrameniscal high signal was in communication with the surface of the meniscus. The pattern of meniscal tears and their location with respect to the vascular zones were classified according to Cooper et al. (1991) (Table).

**Table:** Cliinical data in 25 patients

A	B	C	D	E	F	G	H	I	J	K	L	M	N	O
1	M	14.3	Yes	LM;	Right	R (LM);	all inside	PH (LM);	1	RW (LM);	9	8	96	1.6
				MM		BH (MM)		PI (MM)		RW (MM)				
2	M	16.4	Yes	MM	Left	L	all inside	PH	0	RW	10	10	99	2.3
3	M	15.9	Yes	LM	Right	BH	all inside	PH	0	RR	7	7	99	2.7
4	M	15.3	Yes	LM	Right	L	all inside	PH	0	RW, WW	10	10	99	2.1
5	F	17.3	Yes	MM	Left	H	all inside **^c^**	PH	0	RW	7	5	56	3.7
6	F	15.2	Yes	MM	Right	BH	all inside	PH	2	RR	6	6	96	2
7	F	17.1	Yes	LM;	Right	L (LM);	all inside	PH (LM);	0	RW (LM);	4	4	98	2.7
				MM		BH (MM)		PI (MM)		RW (MM)				
8	M	12.9	No	MM	Left	BH	all inside	PH–PI	0	RW	5	5	95	2.5
9	F	15.5	No	MM	Right	BH	all inside	PH–PI	0	WW	10	9	94	2.4
10	F	17.4	No	MM	Left	BH	all inside	PH	0	RR	9	6	80	2.2
11	F	4.0	No	LM	Right	BH	outside-in	PH	0	RR	9	9	100	1.3
12	M	12.4	No	MM	Left	BH	all inside	PH–PI	0	RW, WW	10	7	90	2.1
13	M	11.9	No	LM	Left	BH	outside-in	PI	0	WW	5	9	93	1.1
14	M	16.2	No	MM	Right	BH	outside-in **^c^**	PI	0	WW	8	5	99	5.1
15	M	15.8	Yes	LM;	Right	BH (LM);	all inside	PI (LM);	1	WW (LM);	7	7	94	1.5
				MM		BH (MM)		PI (MM)		RW (MM)				
16	M	12.5	Yes **^a^**	MM	Right	BH	all inside	PI	0	RR	8	8	100	3.2
17	M	18.5	No	MM	Right	BH	all inside	PI	1	RW	7	5	81	2.5
18	M	15.3	No	MM	Left	L	all inside	PI	1	RW, WW	10	10	100	1.3
19	M	16.9	Yes	LM	Left	L	all inside	PI	3	RW, WW	10	6	99	3.0
20	F	14.4	No	LM	Left	L	all inside	PI	0	RW	7	7	95	2.2
21	F	14.8	No	MM	Left	L	all inside **^c^**	PI	0	RR	5	5	100
22	F	14.2	Yes	LM;	Left	BH (LM);	all inside	PI (LM);	0	RW (LM);	8	8	100	1.8
				MM		BH (MM)		PH (MM)		RW, WW (MM)				
23	F	17.3	No	MM	Right	BH	all inside **^c^**	PI–PH	0	RR	8	7	100	2.2
24	F	11.2	Yes **^b^**	MM	Left	BH	all inside	AH–PI	0	RW, WW	7	7	100	2.8
25	F	11.2	Yes	MM	Right	C	all inside	AH–PI–PH	0	RR	9	9	100	2.2

A.Patient B. Sex C. Age (years) D. ACL-Reconstruction

a after 4.7 years

b after 2.2 years

E. Affected meniscus  MM Medial meniscus  LM Lateral meniscus F. Side G. Tear pattern  BH bucket-handle  L longitudinal  H horizontal  R radial  C complex H. Technique of repair

c Re-rupture

I. Location of tear  PH posterior horn  PI pars intermedia  AH anterior horn J. Chondromalacia (Outerbridge 1961) K. Vascular zone  RR Red–red  RW Red–white  WW White–white L. Tegner score preoperative M. Tegner score postoperative N. Lysholm score at last follow up O. Individual time of follow-up (year)

### Surgical technique

All tears were repaired regardless of the vascular zone in which they occurred (Arnoczky and Warren 1982). A routine arthroscopic setup and standard arthroscopic portals were used to evaluate menisci, articular cartilage, and the intraarticular ligaments. Meniscal tears were prepared by rasping and were anatomically reduced prior to fixation. The methods of fixation were all-inside in 25 cases and an outside-in technique was used in the other 4 cases.

Meniscal sutures were placed vertically on the posterior horn of the lateral meniscus to avoid the popliteus tendon and the peroneal nerve. 1–4 sutures were used for fixation, depending on the size of the tear. Intraoperative findings were recorded using the IKDC protocol.

Simultaneous ACL reconstruction was performed in the presence of an intraligamentous rupture by an arthroscopy-assisted transphyseal technique (Schneider et al. 2008) using free hamstring graft fixed with a biodegradable interference screw (Biosure HA; Smith and Nephew, Austria).

Postoperatively, patients were mobilized non-weight-bearing on forearm crutches and the range of knee motion was restricted to 0–60° in an orthosis for 4 weeks (MEDISAN Sanitätshaus GmbH, Kufstein, Austria). Then the range of motion was increased to 0–90° and the patient progressed to full weight bearing by 6 weeks postoperatively. Reconstruction of the anterior cruciate ligament did not alter the rehabilitation program. Pivoting on a bent, loaded knee was not allowed for the first 3 months. Gradual return to sport was allowed 4–6 months postoperatively, depending on whether the cruciate ligament had also been reconstructed.

### Follow-up

All patients completed a Tegner score pre- and postoperatively and a Lysholm score postoperatively. All postoperative scores were performed as part of a planned clinical follow-up session for this study at a mean of 2.3 (range 1.2–5.1) years postoperatively. Postoperative signs and symptoms such as instability, catching, locking, or swelling were documented. Healing of the menicus was assumed when the patient was clinically symptom-free. The ability to undertake activities of daily living, level of sporting activities, the range of motion of the knee, and the need for reoperation were recorded. Postoperative MRI was performed in 9 knees and arthroscopy was performed in 2 knees.

### Statistics

Paired-samples t-test was used to compare preoperative and postoperative values of Tegner scores and to compare the Lysholm scores between the “meniscus repair” group and the “meniscus with simultaneous ACL reconstruction” group. P-values of < 0.05 were defined as being statistically significant. Results are given as mean (range) with 95% confidence intervals (CIs). We used SPSS software version 16.0.

## Results

### Patient demographics and injury patterns

**Figure 1.**
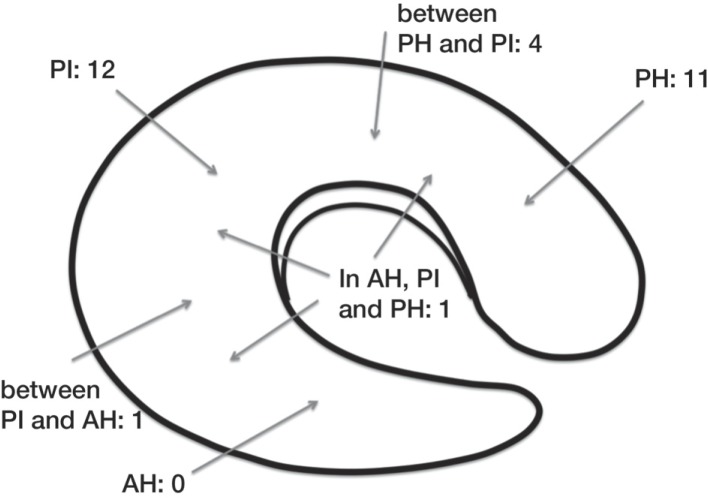
The number of meniscal lesions in each part of the meniscus. Note that most lesions in this age group occur in the pars intermedia and the posterior horn. PH: posterior horn; PI: pars intermedia; AH: anterior horn.

**Figure 2.**
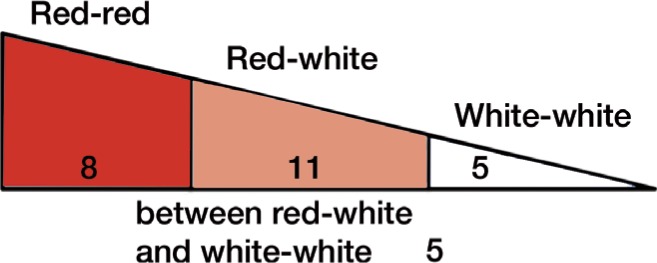
Location of meniscal tears in relation to vascular zones. Most unstable mensical tears in skeletally immature patients were found in the red-red and red-white zone; 5 were found in the white-white zone and 5 were found between these two zones.

We identified 25 patients (13 boys) with 29 meniscal injuries that were eligible for inclusion in the study. All operations were performed by a single surgeon (FS). Average age at the time of operation was 15 (4–17) years. The mean postoperative follow-up was 27 (13–60) months. All meniscal tears were traumatic and none were associated with a discoid meniscus (Table and Figures 1 and 2).

26 menisci either had a longitudinal tear or a bucket-handle tear, 16 in the medial meniscus and 10 in the lateral meniscus. Of the 3 remaining menisci, 1 had an isolated horizontal tear, 1 had a radial tear, and 1 had a complex injury with multiple-plane combination with 2 tear components. 19 of the 26 longitudinal tears were dislocated bucket-handle tears.

13 patients (6 boys) had a concomitant ACL rupture with 6 involving the medial meniscus, 4 the lateral meniscus, and 3 involving both menisci. 11 underwent simultaneous ACL reconstruction with free hamstring autograft. Reconstruction of the ACL was delayed in 2 patients due to their young age (a boy 12.5 years old and a girl 11.2 years old at the time of meniscal repair, both Tanner stage two) (Marshall and Tanner 1969, 1970). ACL reconstruction in these patients was performed 5 years after meniscal repair (the boy) and 2 years after meniscal repair (the girl). This provided an opportunity to examine the repaired menisci, which had healed without any degenerative changes or scarring; they were stable and had a smooth surface without tears or rims. The surrounding articular cartilage was in good condition and there was no evidence of synovitis.

### Outcome

The outcome of meniscal repair was similar for the lateral and medial meniscus. All patients returned to a full level of activity by 6 months after meniscal repair. There was no difference between the patients regarding the zone of meniscal injury and the subsequent method of repair. 4 patients had recurrent meniscal tears following a new injury at mean 15 (7–27) months after the initial meniscal repair. In 2 of the re-ruptures, degeneration of a central part of the meniscus demanded removal. In the other 2 cases, the initially repaired meniscus had to be partially removed but the remaining meniscus had healed and could be preserved. The recurrent tears were all located in the same zone as the original injury. We regarded these 4 re-ruptures as failures of the initial meniscal repair, giving a total failure rate of 16%.

The mean preoperative Tegner score was 7.8 (range 4–10; CI ± 0.75). For the combined repair group, preoperative Tegner score was 7.8 (range 4–10; CI ± 0.96) and postoperative score was 7.2 (4–10; CI ± 0.98). For the “meniscus repair” group, preoperative Tegner score was 7.6 (range 5–10; CI ±1.2) and postoperative score was 7.0 (range 5–10; CI ± 1.1).

Postoperatively, there was a deterioration in the Tegner score without statistical significance (p = 0.05), to a mean of 7.2 (range 4–10; CI ± 0.7). Most patients returned to sports but performed them at a lower level of competitiveness—by 1 or 3 points; 1 boy with combined repair even reduced his competitiveness level by 4 points: from level 9 (Tegner) to level 5 (Tegner). Reduction of competitiveness level was due to fear of a new injury.

The mean postoperative Lysholm score was 95 (81–100) points for the whole cohort. The main problems in patients with low Lysholm scores were pain and swelling during severe exertion and also impaired squatting. The Lysholm scores of the ACL-reconstructed group and the ACL-intact group were similar (95 and 94).

## Discussion

The true incidence of meniscal injuries in children is unknown, but with increasing participation in competitive sports the number of knee injuries will inevitably rise. There are a multitude of reports on the results of repairing meniscal tears in adults or in a mixed population of adults and adolescents. In this population, the success of meniscal repair depends on the type of tear, location within the meniscus (particularly with regard to the vascular zone), tear length, rim width, and other factors (Bach et al. 2005, Kimura et al. 1995, Tenuta and Arciero 1994, Scott et al. 1986). Success rates for meniscal repairs in these groups are reported to be good to fair (Barber 1987, Eggli et al. 1995). Mintzer et al. (1998) reported 26 patients who were all asymptomatic at a follow-up after 5 years, and Anderson (2003) confirmed excellent outcomes from simultaneous ACL repair (8/12) at a follow-up time of 4 years. In a more recent study, Vanderhave et al. (year) reported 43 of 45 menisci to be clinically healed 2 years after meniscal repair.

We repaired all meniscal tears regardless of their location or pattern in the hope of preserving meniscal function, and to avoid predisposing factors for early osteoarthritis (Fairbank 1948). Partial resection of the meniscus was reserved only for those cases in which the initial repair had failed. Meniscal repair was successful in 25 of 29 menisci after more than 2 years. In the unsuccessful cases, the patients had sustained meniscal tears during sporting activity and were asymptomatic prior to the re-injury.

Primary healing of meniscal tears depends on location and vascularity (Arnoczky and Warren 1982, Cooper et al. 1991) and also on tear pattern and meniscal quality. It is generally believed that poor vascular supply limits the healing capability of the central zones (Arnoczky and Warren 1982). The results of our study and those of others suggest that the repair of meniscal tears in children and adolescents is successful regardless of their location and of the vascular zone (Noyes and Barber-Westin 2002, Bach et al. 2005, Lorbach et al. 2009). In the adult meniscus, only the peripheral one-third is vascularized, whereas at birth the meniscus in its entirety is vascular and in children these vessels can still be identified throughout the inner zones (Clark and Ogden 1983).

Animal explant culture models have shown meniscal tissue to be capable of a repair response in the absence of vascularization (Hennerbichler et al. 2007). Kalliakmanis et al. (2008) did not find any significant difference between tears located in the red-on-red zone and those in the red-on-white zone, and Rubman et al. (1998) found 80% to be asymptomatic following repair of meniscal tears that extended into the avascular zone, even in adults.

As the anterior and posterior horns of the menisci are better vascularized than the pars intermedia (Arnoczky and Warren 1982, Danzig et al. 1983, Day et al. 1985), healing in these zones is therefore thought to be more successful. In 15 of our patients, the meniscal tears were in or extended into the pars intermedia; however, all healed primarily. 4 patients sustained recurrent tears. All were located in the pars intermedia. Despite initial healing, we conclude that the quality of healing in this zone may be inferior.

Healing of meniscal tears also depends on their pattern (Laprell et al. 2002, Haas et al. 2005) with longitudinal tears that have intact circumferential fibers showing better healing potential (Messner et al. 1998). Longitudinal and bucket-handle meniscal tears are strongly associated with ACL ruptures. We noted an ACL rupture in 13 patients, which were all associated with either a bucket-handle tear or a longitudinal meniscal tear. It is interesting that the pattern of meniscal tears in children tend to be longitudinal peripheral or bucket-handle in the red-red meniscal zone. As children have relatively lax joints (Kirk et al. 1967), their menisci are subjected to enhanced shearing forces at the menisco-synovial junction, such that longitudinal tears would leave the circumferential meniscal fibers intact. Radial tears, in contrast, are very rare in children, and are seen much more frequently in the adult population in association with a degenerate meniscus.

Different suture techniques did not affect healing in our patients. All-inside bioresorbable fixation and outside-in fixation are known to produce similar results (Kalliakmanis et al. 2008, Choi et al. 2009) and all-inside fixation is also a safe procedure in children (Ahn et al. 2004).

Half also had an ACL rupture. DeHaven (1990) reported that 80% of their patients with a meniscal tear also had an anterior cruciate ligament lesion. It has been reported that patients with simultaneous repair of the meniscus and anterior cruciate ligament have more favorable outcome (Cannon and Vittori 1992, Tenuta and Arciero 1994) with higher healing rates (Jensen et al. 1994, Tenuta and Arciero 1994, Kimura et al. 1995). Bleeding induced by ACL reconstruction creates a blood clot at the site of the meniscal suture. This provides a supply of bone marrow stem cells (BMSCs) (Abdel-Hamid et al. 2005) that may be beneficial to the healing process (Cannon and Arciero 1992, Jensen et al. 1994). For this reason, we favor the simultaneous repair of these combined injuries when the age of the patient permits.

Initially, our postoperative follow-up protocol included MRI scanning 3 months after surgery. This was only performed in 9 patients and was subsequently discontinued due to the non-specific nature of the findings and high costs. In 3 cases, the scans were essentially normal with healed menisci. The other 6 showed alterations ranging from suspected re-rupture to mucoid degeneration, but the patients were asymptomatic. The discrepancy between clinical and MRI findings was reported by Muellner et al. (1999), where half of his patients showed grade-3 signal changes at a mean of 13 years after meniscal repair, but none had any associated clinical symptoms. Similarly, in a study by Eggli et al. (1995) 96% of successfully repaired menisci in asymptomatic patients showed a persistent grade-3 or grade-4 lesion (Eggli et al. 1995) on MRI. In a more recent study with 13 years of follow-up, Steenbrugge et al. (2004) described mucoid degeneration or scarring in 6 of 13 patients, although they all had good long-term clinical results.

Our study had some limitations. It was retrospective, the number of patients was relatively small, and the follow-up period was short. All of our patients had improvement of symptoms after surgery, but only a limited number had a postoperative MRI scan or were re-evaluated by arthroscopy during delayed ACL reconstruction. Absence of symptoms is not equivalent to meniscal healing, which can only be confirmed arthroscopically (Tenuta and Arciero 1994). Only 6 of our patients had an arthroscopic evaluation, for whom there was an indication because of new trauma or delayed ACL reconstruction. Due to the short follow-up time, our results must be interpreted with caution because it is well documented that outcomes deteriorate with time (McNicholas et al. 2000).
